# Supplementary material for: Performance of Wearable Pulse Oximetry During Controlled Hypoxia Induction: Instrument Validation Study
Source: JMIR Form Res. 2026 Mar 27;10:e85253. doi: 10.2196/85253 (PMC13026435; doi:10.2196/85253)
Supplement: Multimedia Appendix 1 [file formative-v10-e85253-s001.docx]

| Participant Number | MDE ± SD of all measurements from Apple Watch (%) | MDE ± SD of all measurements from Masimo MightySat Rx (%) | MDE ± SD of  Hypoxemia-only measurements from Apple Watch (%) | MDE ± SD of Hypoxemia-only measurements from Masimo MightySat Rx (%) |
| --- | --- | --- | --- | --- |
| 1 | 1.13 ± 2.00 | 0.50 ± 1.39 | 2.26 ± 1.46 | 0.26 ± 1.72 |
| 2 | 5.78 ± 4.20 | 2.93 ± 1.91 | 8.77 ± 2.51 | 3.49 ± 2.02 |
| 3 | 3.15 ± 4.07 | 1.47 ± 1.75 | 6.51 ± 2.76 | 1.74 ± 1.82 |
| 4 | 2.34 ± 1.41 | 2.56 ± 2.26 | 3.16 ± 1.18 | 3.97 ± 1.73 |
| 5 | 1.76 ± 1.13 | 3.09 ± 2.06 | 2.29 ± 1.19 | 3.79 ± 2.33 |
| 6 | 2.95 ± 2.96 | -0.77 ± 2.29 | 4.47 ± 2.36 | -1.93 ± 2.39 |
| 7 | 3.13 ± 5.28 | 2.82 ± 3.00 | 8.24 ± 2.96 | 2.93 ± 3.82 |
| 8 | 4.80 ± 2.59 | 1.66 ± 1.22 | 6.74 ± 1.49 | 1.53 ± 1.50 |
| 9 | 4.63 ± 2.58 | 1.66 ± 2.69 | 5.99 ± 2.14 | 1.22 ± 3.44 |
