# Supplementary figures and images for: Performance of Wearable Pulse Oximetry During Controlled Hypoxia Induction: Instrument Validation Study
Source: JMIR Form Res. 2026 Mar 27;10:e85253. doi: 10.2196/85253 (PMC13026435; doi:10.2196/85253)

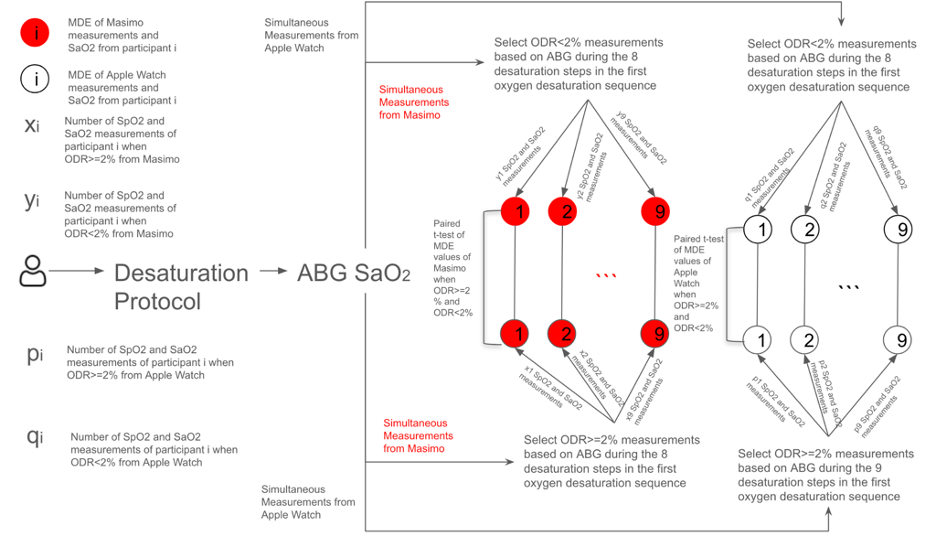

Supplement: Multimedia Appendix 2 [file formative-v10-e85253-s002.png]

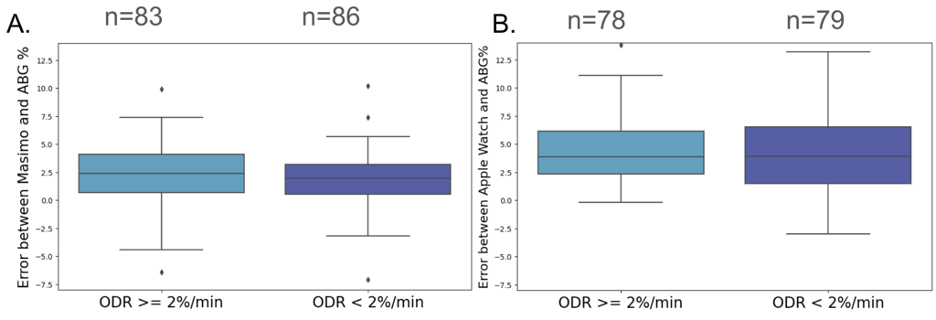

Supplement: Multimedia Appendix 3 [file formative-v10-e85253-s003.png]

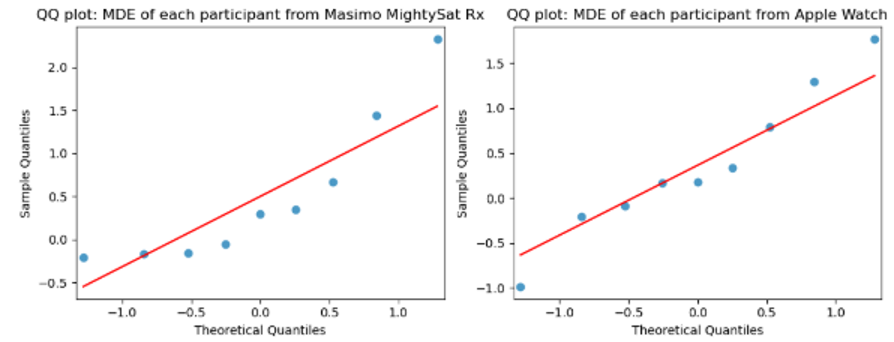

Supplement: Multimedia Appendix 4 [file formative-v10-e85253-s004.png]

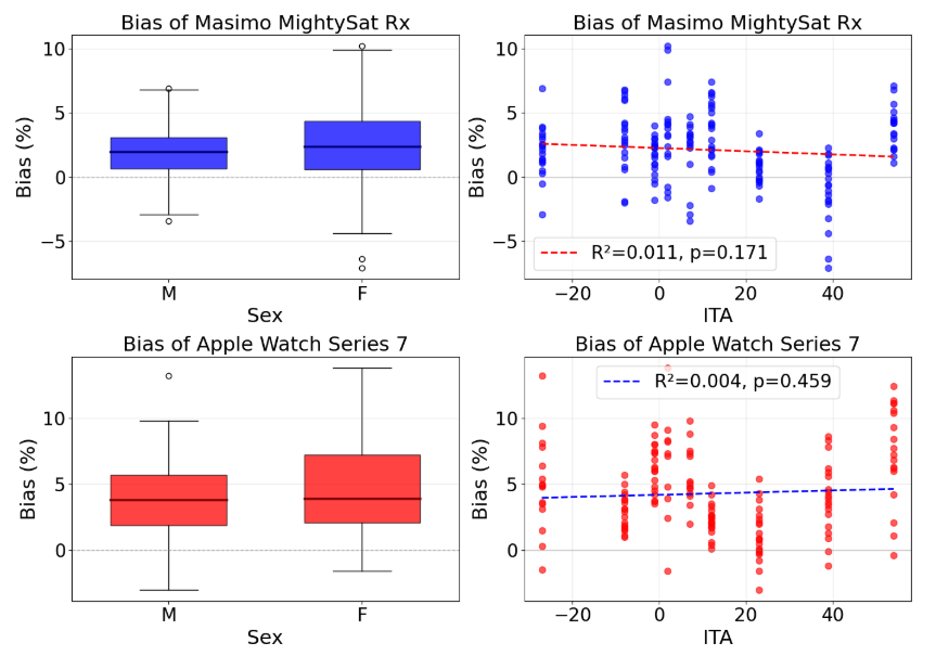

Supplement: Multimedia Appendix 6 [file formative-v10-e85253-s006.png]
