# Supplementary material for: Performance of Wearable Pulse Oximetry During Controlled Hypoxia Induction: Instrument Validation Study
Source: JMIR Form Res. 2026 Mar 27;10:e85253. doi: 10.2196/85253 (PMC13026435; doi:10.2196/85253)
Supplement: Multimedia Appendix 5 [file formative-v10-e85253-s005.docx]

**Supplementary Table 2.** SpO_2_ and SaO_2_ measurements when ODR >=2% or ODR < 2%

| Device | ODR_category | Subject | SpO_2_ | SaO_2_ | Bias (SpO_2_ -SaO_2_ ) | MDE (%) |
| --- | --- | --- | --- | --- | --- | --- |
| Apple Watch | ODR>=2% | 69 | 94 | 90.9 | 3.1 | 2.2375 |
|  |  | 69 | 88 | 82.6 | 5.4 |  |
|  |  | 69 | 80 | 77.7 | 2.3 |  |
|  |  | 69 | 75 | 71.2 | 3.8 |  |
|  |  | 69 | 91 | 91.2 | -0.2 |  |
|  |  | 69 | 86 | 85.9 | 0.1 |  |
|  |  | 69 | 81 | 80.2 | 0.8 |  |
|  |  | 69 | 77 | 74.4 | 2.6 |  |
|  |  | 70 | 90 | 83.7 | 6.3 | 7.42857143 |
|  |  | 70 | 81 | 71.7 | 9.3 |  |
|  |  | 70 | 74 | 63.6 | 10.4 |  |
|  |  | 70 | 98 | 92 | 6 |  |
|  |  | 70 | 94 | 87.2 | 6.8 |  |
|  |  | 70 | 96 | 84.9 | 11.1 |  |
|  |  | 70 | 80 | 77.9 | 2.1 |  |
|  |  | 71 | 93 | 89.9 | 3.1 | 5.21428571 |
|  |  | 71 | 86 | 82.4 | 3.6 |  |
|  |  | 71 | 83 | 76.6 | 6.4 |  |
|  |  | 71 | 95 | 90.1 | 4.9 |  |
|  |  | 71 | 87 | 82 | 5 |  |
|  |  | 71 | 88 | 82.6 | 5.4 |  |
|  |  | 71 | 85 | 76.9 | 8.1 |  |
|  |  | 72 | 91 | 90 | 1 | 3.21818182 |
|  |  | 72 | 86 | 82.2 | 3.8 |  |
|  |  | 72 | 81 | 77.9 | 3.1 |  |
|  |  | 72 | 75 | 69.3 | 5.7 |  |
|  |  | 72 | 65 | 60.6 | 4.4 |  |
|  |  | 72 | 61 | 56 | 5 |  |
|  |  | 72 | 91 | 89.1 | 1.9 |  |
|  |  | 72 | 86 | 83.1 | 2.9 |  |
|  |  | 72 | 85 | 83.3 | 1.7 |  |
|  |  | 72 | 79 | 75.2 | 3.8 |  |
|  |  | 72 | 71 | 68.9 | 2.1 |  |
|  |  | 73 | 93 | 91 | 2 | 2.18333333 |
|  |  | 73 | 88 | 84.5 | 3.5 |  |
|  |  | 73 | 82 | 79.6 | 2.4 |  |
|  |  | 73 | 75 | 72.4 | 2.6 |  |
|  |  | 73 | 69 | 67.2 | 1.8 |  |
|  |  | 73 | 66 | 62.7 | 3.3 |  |
|  |  | 73 | 62 | 57.8 | 4.2 |  |
|  |  | 73 | 93 | 91.3 | 1.7 |  |
|  |  | 73 | 86 | 84.2 | 1.8 |  |
|  |  | 73 | 87 | 84.8 | 2.2 |  |
|  |  | 73 | 81 | 80.4 | 0.6 |  |
|  |  | 73 | 73 | 72.9 | 0.1 |  |
|  |  | 74 | 90 | 90.1 | -0.1 | 4.01818182 |
|  |  | 74 | 86 | 85.1 | 0.9 |  |
|  |  | 74 | 84 | 80.4 | 3.6 |  |
|  |  | 74 | 80 | 73.8 | 6.2 |  |
|  |  | 74 | 71 | 62.4 | 8.6 |  |
|  |  | 74 | 66 | 60.4 | 5.6 |  |
|  |  | 74 | 92 | 90.7 | 1.3 |  |
|  |  | 74 | 86 | 84.2 | 1.8 |  |
|  |  | 74 | 86 | 82.6 | 3.4 |  |
|  |  | 74 | 83 | 78.4 | 4.6 |  |
|  |  | 74 | 85 | 76.7 | 8.3 |  |
|  |  | 75 | 95 | 90.2 | 4.8 | 6.85 |
|  |  | 75 | 85 | 71.2 | 13.8 |  |
|  |  | 75 | 93 | 90.6 | 2.4 |  |
|  |  | 75 | 88 | 84.1 | 3.9 |  |
|  |  | 75 | 91 | 83.9 | 7.1 |  |
|  |  | 75 | 88 | 78.9 | 9.1 |  |
|  |  | 76 | 94 | 89.5 | 4.5 | 6.13333333 |
|  |  | 76 | 88 | 84.3 | 3.7 |  |
|  |  | 76 | 85 | 77.6 | 7.4 |  |
|  |  | 76 | 79 | 71 | 8 |  |
|  |  | 76 | 69 | 61.5 | 7.5 |  |
|  |  | 76 | 94 | 90.5 | 3.5 |  |
|  |  | 76 | 90 | 84.7 | 5.3 |  |
|  |  | 76 | 91 | 83.7 | 7.3 |  |
|  |  | 76 | 81 | 73 | 8 |  |
|  |  | 77 | 94 | 86.7 | 7.3 | 5.14285714 |
|  |  | 77 | 74 | 68.9 | 5.1 |  |
|  |  | 77 | 94 | 89.9 | 4.1 |  |
|  |  | 77 | 89 | 81.9 | 7.1 |  |
|  |  | 77 | 87 | 83.6 | 3.4 |  |
|  |  | 77 | 82 | 77.3 | 4.7 |  |
|  |  | 77 | 74 | 69.7 | 4.3 |  |
|  | ODR<2% | 69 | 98 | 97.1 | 0.9 | 0.46923077 |
|  |  | 69 | 88 | 88 | 0 |  |
|  |  | 69 | 89 | 86.9 | 2.1 |  |
|  |  | 69 | 78 | 77.5 | 0.5 |  |
|  |  | 69 | 75 | 70.7 | 4.3 |  |
|  |  | 69 | 72 | 70 | 2 |  |
|  |  | 69 | 96 | 96.8 | -0.8 |  |
|  |  | 69 | 98 | 97.2 | 0.8 |  |
|  |  | 69 | 95 | 96.6 | -1.6 |  |
|  |  | 69 | 91 | 91.1 | -0.1 |  |
|  |  | 69 | 88 | 91 | -3 |  |
|  |  | 69 | 85 | 85.3 | -0.3 |  |
|  |  | 69 | 86 | 84.7 | 1.3 |  |
|  |  | 70 | 93 | 91.9 | 1.1 | 7.25454545 |
|  |  | 70 | 91 | 91.4 | -0.4 |  |
|  |  | 70 | 93 | 82.4 | 10.6 |  |
|  |  | 70 | 86 | 78.8 | 7.2 |  |
|  |  | 70 | 76 | 64.7 | 11.3 |  |
|  |  | 70 | 75 | 62.6 | 12.4 |  |
|  |  | 70 | 98 | 91.8 | 6.2 |  |
|  |  | 70 | 94 | 89.8 | 4.2 |  |
|  |  | 70 | 92 | 83.6 | 8.4 |  |
|  |  | 70 | 90 | 82.3 | 7.7 |  |
|  |  | 70 | 88 | 76.9 | 11.1 |  |
|  |  | 71 | 95 | 96.5 | -1.5 | 4.87777778 |
|  |  | 71 | 86 | 81.2 | 4.8 |  |
|  |  | 71 | 98 | 96.5 | 1.5 |  |
|  |  | 71 | 97 | 96.7 | 0.3 |  |
|  |  | 71 | 93 | 89.5 | 3.5 |  |
|  |  | 71 | 87 | 82.1 | 4.9 |  |
|  |  | 71 | 84 | 76.2 | 7.8 |  |
|  |  | 71 | 83 | 73.6 | 9.4 |  |
|  |  | 71 | 85 | 71.8 | 13.2 |  |
|  |  | 72 | 98 | 96.9 | 1.1 | 2.425 |
|  |  | 72 | 98 | 96.4 | 1.6 |  |
|  |  | 72 | 91 | 89.2 | 1.8 |  |
|  |  | 72 | 80 | 77.5 | 2.5 |  |
|  |  | 72 | 76 | 72.4 | 3.6 |  |
|  |  | 72 | 71 | 66.8 | 4.2 |  |
|  |  | 72 | 98 | 96.5 | 1.5 |  |
|  |  | 72 | 91 | 87.9 | 3.1 |  |
|  |  | 73 | 93 | 90.9 | 2.1 | 2.38888889 |
|  |  | 73 | 82 | 79 | 3 |  |
|  |  | 73 | 74 | 71.6 | 2.4 |  |
|  |  | 73 | 65 | 62.4 | 2.6 |  |
|  |  | 73 | 62 | 57.1 | 4.9 |  |
|  |  | 73 | 99 | 96.9 | 2.1 |  |
|  |  | 73 | 86 | 84.6 | 1.4 |  |
|  |  | 73 | 82 | 79.4 | 2.6 |  |
|  |  | 73 | 73 | 72.6 | 0.4 |  |
|  |  | 74 | 89 | 84.6 | 4.4 | 3.8375 |
|  |  | 74 | 83 | 78.9 | 4.1 |  |
|  |  | 74 | 76 | 72.1 | 3.9 |  |
|  |  | 74 | 77 | 69.2 | 7.8 |  |
|  |  | 74 | 66 | 60.1 | 5.9 |  |
|  |  | 74 | 99 | 96.3 | 2.7 |  |
|  |  | 74 | 95 | 96.2 | -1.2 |  |
|  |  | 74 | 82 | 78.9 | 3.1 |  |
|  |  | 75 | 95 | 96.6 | -1.6 | 5.55 |
|  |  | 75 | 92 | 83.8 | 8.2 |  |
|  |  | 75 | 92 | 83.7 | 8.3 |  |
|  |  | 75 | 86 | 78.7 | 7.3 |  |
|  |  | 76 | 99 | 95.3 | 3.7 | 6.22 |
|  |  | 76 | 89 | 83 | 6 |  |
|  |  | 76 | 83 | 76.7 | 6.3 |  |
|  |  | 76 | 79 | 70.3 | 8.7 |  |
|  |  | 76 | 71 | 66.8 | 4.2 |  |
|  |  | 76 | 72 | 65.8 | 6.2 |  |
|  |  | 76 | 68 | 61.2 | 6.8 |  |
|  |  | 76 | 95 | 90.2 | 4.8 |  |
|  |  | 76 | 90 | 84 | 6 |  |
|  |  | 76 | 93 | 83.5 | 9.5 |  |
|  |  | 77 | 94 | 86.5 | 7.5 | 6.12857143 |
|  |  | 77 | 90 | 81.2 | 8.8 |  |
|  |  | 77 | 77 | 67.2 | 9.8 |  |
|  |  | 77 | 66 | 64 | 2 |  |
|  |  | 77 | 95 | 89.8 | 5.2 |  |
|  |  | 77 | 95 | 90.1 | 4.9 |  |
|  |  | 77 | 74 | 69.3 | 4.7 |  |
| Masimo | ODR>=2% | 69 | 92 | 90.9 | 1.1 | 0.6125 |
|  |  | 69 | 86 | 82.6 | 3.4 |  |
|  |  | 69 | 76 | 77.7 | -1.7 |  |
|  |  | 69 | 73 | 71.2 | 1.8 |  |
|  |  | 69 | 91 | 91.2 | -0.2 |  |
|  |  | 69 | 87 | 85.9 | 1.1 |  |
|  |  | 69 | 80 | 80.2 | -0.2 |  |
|  |  | 69 | 74 | 74.4 | -0.4 |  |
|  |  | 70 | 88 | 83.7 | 4.3 | 4.28571429 |
|  |  | 70 | 75 | 71.7 | 3.3 |  |
|  |  | 70 | 68 | 63.6 | 4.4 |  |
|  |  | 70 | 95 | 92 | 3 |  |
|  |  | 70 | 94 | 87.2 | 6.8 |  |
|  |  | 70 | 92 | 84.9 | 7.1 |  |
|  |  | 70 | 79 | 77.9 | 1.1 |  |
|  |  | 71 | 91 | 89.9 | 1.1 | 2.8375 |
|  |  | 71 | 85 | 82.4 | 2.6 |  |
|  |  | 71 | 80 | 76.6 | 3.4 |  |
|  |  | 71 | 71 | 69.8 | 1.2 |  |
|  |  | 71 | 97 | 90.1 | 6.9 |  |
|  |  | 71 | 85 | 82 | 3 |  |
|  |  | 71 | 85 | 82.6 | 2.4 |  |
|  |  | 71 | 79 | 76.9 | 2.1 |  |
|  |  | 72 | 88 | 90 | -2 | 4.12727273 |
|  |  | 72 | 86 | 82.2 | 3.8 |  |
|  |  | 72 | 82 | 77.9 | 4.1 |  |
|  |  | 72 | 76 | 69.3 | 6.7 |  |
|  |  | 72 | 67 | 60.6 | 6.4 |  |
|  |  | 72 | 62 | 56 | 6 |  |
|  |  | 72 | 91 | 89.1 | 1.9 |  |
|  |  | 72 | 86 | 83.1 | 2.9 |  |
|  |  | 72 | 86 | 83.3 | 2.7 |  |
|  |  | 72 | 82 | 75.2 | 6.8 |  |
|  |  | 72 | 75 | 68.9 | 6.1 |  |
|  |  | 73 | 95 | 91 | 4 | 3.85 |
|  |  | 73 | 91 | 84.5 | 6.5 |  |
|  |  | 73 | 85 | 79.6 | 5.4 |  |
|  |  | 73 | 79 | 72.4 | 6.6 |  |
|  |  | 73 | 73 | 67.2 | 5.8 |  |
|  |  | 73 | 69 | 62.7 | 6.3 |  |
|  |  | 73 | 62 | 57.8 | 4.2 |  |
|  |  | 73 | 93 | 91.3 | 1.7 |  |
|  |  | 73 | 88 | 84.2 | 3.8 |  |
|  |  | 73 | 87 | 84.8 | 2.2 |  |
|  |  | 73 | 81 | 80.4 | 0.6 |  |
|  |  | 73 | 72 | 72.9 | -0.9 |  |
|  |  | 74 | 90 | 90.1 | -0.1 | -1.1636364 |
|  |  | 74 | 83 | 85.1 | -2.1 |  |
|  |  | 74 | 79 | 80.4 | -1.4 |  |
|  |  | 74 | 72 | 73.8 | -1.8 |  |
|  |  | 74 | 58 | 62.4 | -4.4 |  |
|  |  | 74 | 54 | 60.4 | -6.4 |  |
|  |  | 74 | 91 | 90.7 | 0.3 |  |
|  |  | 74 | 85 | 84.2 | 0.8 |  |
|  |  | 74 | 82 | 82.6 | -0.6 |  |
|  |  | 74 | 79 | 78.4 | 0.6 |  |
|  |  | 74 | 79 | 76.7 | 2.3 |  |
|  |  | 75 | 94 | 90.2 | 3.8 | 3.5375 |
|  |  | 75 | 81 | 79.2 | 1.8 |  |
|  |  | 75 | 70 | 71.2 | -1.2 |  |
|  |  | 75 | 64 | 65.6 | -1.6 |  |
|  |  | 75 | 98 | 90.6 | 7.4 |  |
|  |  | 75 | 94 | 84.1 | 9.9 |  |
|  |  | 75 | 88 | 83.9 | 4.1 |  |
|  |  | 75 | 83 | 78.9 | 4.1 |  |
|  |  | 76 | 92 | 89.5 | 2.5 | 1.82 |
|  |  | 76 | 86 | 84.3 | 1.7 |  |
|  |  | 76 | 80 | 77.6 | 2.4 |  |
|  |  | 76 | 72 | 71 | 1 |  |
|  |  | 76 | 61 | 61.5 | -0.5 |  |
|  |  | 76 | 92 | 90.5 | 1.5 |  |
|  |  | 76 | 87 | 84.7 | 2.3 |  |
|  |  | 76 | 87 | 83.7 | 3.3 |  |
|  |  | 76 | 82 | 79 | 3 |  |
|  |  | 76 | 74 | 73 | 1 |  |
|  |  | 77 | 90 | 86.7 | 3.3 | 1.825 |
|  |  | 77 | 70 | 73.4 | -3.4 |  |
|  |  | 77 | 66 | 68.9 | -2.9 |  |
|  |  | 77 | 93 | 89.9 | 3.1 |  |
|  |  | 77 | 86 | 81.9 | 4.1 |  |
|  |  | 77 | 87 | 83.6 | 3.4 |  |
|  |  | 77 | 82 | 77.3 | 4.7 |  |
|  |  | 77 | 72 | 69.7 | 2.3 |  |
|  | ODR<2% | 69 | 99 | 97.1 | 1.9 | 0.77692308 |
|  |  | 69 | 89 | 88 | 1 |  |
|  |  | 69 | 89 | 86.9 | 2.1 |  |
|  |  | 69 | 78 | 77.5 | 0.5 |  |
|  |  | 69 | 70 | 70.7 | -0.7 |  |
|  |  | 69 | 72 | 70 | 2 |  |
|  |  | 69 | 98 | 96.8 | 1.2 |  |
|  |  | 69 | 98 | 97.2 | 0.8 |  |
|  |  | 69 | 97 | 96.6 | 0.4 |  |
|  |  | 69 | 91 | 91.1 | -0.1 |  |
|  |  | 69 | 91 | 91 | 0 |  |
|  |  | 69 | 85 | 85.3 | -0.3 |  |
|  |  | 69 | 86 | 84.7 | 1.3 |  |
|  |  | 70 | 94 | 91.9 | 2.1 | 3.61818182 |
|  |  | 70 | 93 | 91.4 | 1.6 |  |
|  |  | 70 | 87 | 82.4 | 4.6 |  |
|  |  | 70 | 83 | 78.8 | 4.2 |  |
|  |  | 70 | 67 | 64.7 | 2.3 |  |
|  |  | 70 | 66 | 62.6 | 3.4 |  |
|  |  | 70 | 96 | 91.8 | 4.2 |  |
|  |  | 70 | 92 | 89.8 | 2.2 |  |
|  |  | 70 | 88 | 83.6 | 4.4 |  |
|  |  | 70 | 88 | 82.3 | 5.7 |  |
|  |  | 70 | 82 | 76.9 | 5.1 |  |
|  |  | 71 | 97 | 96.5 | 0.5 | 1.39090909 |
|  |  | 71 | 85 | 81.2 | 3.8 |  |
|  |  | 71 | 77 | 75.7 | 1.3 |  |
|  |  | 71 | 72 | 74.9 | -2.9 |  |
|  |  | 71 | 96 | 96.5 | -0.5 |  |
|  |  | 71 | 97 | 96.7 | 0.3 |  |
|  |  | 71 | 92 | 89.5 | 2.5 |  |
|  |  | 71 | 86 | 82.1 | 3.9 |  |
|  |  | 71 | 78 | 76.2 | 1.8 |  |
|  |  | 71 | 75 | 73.6 | 1.4 |  |
|  |  | 71 | 75 | 71.8 | 3.2 |  |
|  |  | 72 | 95 | 96.9 | -1.9 | 1.8 |
|  |  | 72 | 97 | 96.4 | 0.6 |  |
|  |  | 72 | 90 | 89.2 | 0.8 |  |
|  |  | 72 | 80 | 77.5 | 2.5 |  |
|  |  | 72 | 76 | 72.4 | 3.6 |  |
|  |  | 72 | 71 | 66.8 | 4.2 |  |
|  |  | 72 | 98 | 96.5 | 1.5 |  |
|  |  | 72 | 91 | 87.9 | 3.1 |  |
|  |  | 73 | 95 | 90.9 | 4.1 | 3.5 |
|  |  | 73 | 84 | 79 | 5 |  |
|  |  | 73 | 79 | 71.6 | 7.4 |  |
|  |  | 73 | 68 | 62.4 | 5.6 |  |
|  |  | 73 | 60 | 57.1 | 2.9 |  |
|  |  | 73 | 99 | 96.9 | 2.1 |  |
|  |  | 73 | 87 | 84.6 | 2.4 |  |
|  |  | 73 | 81 | 79.4 | 1.6 |  |
|  |  | 73 | 73 | 72.6 | 0.4 |  |
|  |  | 74 | 97 | 96.3 | 0.7 | -0.9555556 |
|  |  | 74 | 84 | 84.6 | -0.6 |  |
|  |  | 74 | 80 | 78.9 | 1.1 |  |
|  |  | 74 | 71 | 72.1 | -1.1 |  |
|  |  | 74 | 66 | 69.2 | -3.2 |  |
|  |  | 74 | 53 | 60.1 | -7.1 |  |
|  |  | 74 | 98 | 96.3 | 1.7 |  |
|  |  | 74 | 98 | 96.2 | 1.8 |  |
|  |  | 74 | 77 | 78.9 | -1.9 |  |
|  |  | 75 | 99 | 96.6 | 2.4 | 3.5875 |
|  |  | 75 | 87 | 83.8 | 3.2 |  |
|  |  | 75 | 87 | 83.7 | 3.3 |  |
|  |  | 75 | 70 | 70.8 | -0.8 |  |
|  |  | 75 | 98 | 96.4 | 1.6 |  |
|  |  | 75 | 94 | 83.8 | 10.2 |  |
|  |  | 75 | 88 | 83.5 | 4.5 |  |
|  |  | 75 | 83 | 78.7 | 4.3 |  |
|  |  | 76 | 98 | 95.3 | 2.7 | 1.52 |
|  |  | 76 | 86 | 83 | 3 |  |
|  |  | 76 | 79 | 76.7 | 2.3 |  |
|  |  | 76 | 71 | 70.3 | 0.7 |  |
|  |  | 76 | 67 | 66.8 | 0.2 |  |
|  |  | 76 | 64 | 65.8 | -1.8 |  |
|  |  | 76 | 61 | 61.2 | -0.2 |  |
|  |  | 76 | 92 | 90.2 | 1.8 |  |
|  |  | 76 | 88 | 84 | 4 |  |
|  |  | 76 | 86 | 83.5 | 2.5 |  |
|  |  | 77 | 89 | 86.5 | 2.5 | 1.98571429 |
|  |  | 77 | 84 | 81.2 | 2.8 |  |
|  |  | 77 | 65 | 67.2 | -2.2 |  |
|  |  | 77 | 65 | 64 | 1 |  |
|  |  | 77 | 93 | 89.8 | 3.2 |  |
|  |  | 77 | 94 | 90.1 | 3.9 |  |
|  |  | 77 | 72 | 69.3 | 2.7 |  |
